# Gait screening of a population of young, healthy athletes by means of a portable, low-cost device unveils hidden left–right asymmetries in both quadriceps and anterior cruciate ligament forces

**DOI:** 10.1186/s13104-019-4406-x

**Published:** 2019-06-28

**Authors:** Nicolò Colombo, Francesca Vignaga, Eleonora Solari, Mattia Merlo, Alessandro Manelli, Daniela Negrini, Andrea Moriondo

**Affiliations:** 1 Department of Medicine and Surgery, School of Medicine, University of Insubria, Via Monte Generoso, 71, 21100 Varese, Italy; 2 Physiatric Division, ASL 1 Imperiese, Bussana di Sanremo, Italy; 3 School of Medicine, University of Insubria, Varese, Italy; 4 University of Genoa, Genoa, Italy

**Keywords:** Wii balance board, Gait analysis, Population screening, On-field data acquisition

## Abstract

**Objective:**

The present study reports the on-field screening of a population of young soccer players in the pursuit of alterations in gait using a portable and low-cost gait analysis system composed of a Wii Balance Board and a webcam.

**Results:**

Recordings of motion of the lower extremities along with vertical ground reaction force (GRF) were used to quantify coefficients of symmetry for the overall GRF and the forces exerted by the *quadriceps femori* and acting on the anterior cruciate ligament (ACL). Data show that, in face of a quite homogeneous symmetry of GRF during left and right stance phases of gait, quadriceps and ACL exert and are subjected to left–right asymmetrical forces that might prelude, especially in young athletes, later alterations of gait.

## Introduction

Gait analysis is a valuable tool in rehabilitation and allows the physiotherapist to evaluate the physiological and pathological gait in many rehabilitative areas such as neurological and orthopedics, in patients undergoing cruciate ligament surgery, hip or knee prosthesis.

Although gait analysis provides, among others, multiple data regarding body segments movements and ground reaction forces (GRFs), the facilities possessing available equipment, are not widely present due to the need of a dedicated room, personnel and the high costs associated to the purchase of the necessary hardware.

Low cost approaches based on the use of Wii Balance Board (WBB), wooden platforms and web cameras have been successfully used in the past with excellent results if compared to professional solutions. Regarding the vertical component of GRF and sagittal plane recording of ankle, knee and hip markers, a good agreement in terms of GRF values and their standard deviation had been found (about 70 N for WBB-webcam setup, 40 N for commercial system), while the trajectories of body markers were reported as substantially accurate as the ones tracked by a commercial system [1, 2].

Therefore, this study aims to evaluate the on-field use of a WBB and video-recording system in a screening of healthy young soccer players in the pursuit of hidden gait abnormalities.

## Main text

### Methods

The research protocol used in the present work was outside of the definitions of EU 536/2014 directive, regarding projects requiring ethical approval. Data recording, analysis, storage and divulgation followed EU 679/2016 (GDPR) rule and were made in accordance with the Guidelines of the University of Insubria regarding data privacy protection.

A total of 17 athletes practicing agonistic sports (regional soccer championships, 5 males and 12 females, mean age 22.8 ± 3.5, range 17–30), were recruited at the end of the regular season and did not report any problem with walking.

The equipment consisted of a modular and transportable wooden platform 450 cm long × 100 cm wide × 5 cm tall housing a *Nintendo*^*®*^ *Wii Balance Board* (WBB) in a hole measuring 51 cm × 32 cm × 5.3 cm, aligned with the top of the platform.

On a wall parallel and close to the longitudinal axis of the platform, reference markers of size 2 cm × 2 cm were placed at 105 cm from each other for video analysis.

A *webcam* (Logitech, 640 × 480 pixels, 30 fps) was placed laterally at 2.5 m from the side of the platform, aligned with the WBB center and at the same knee height of the subject standing on the WBB.

Recordings of video and WBB data were carried out at the Sports Campus of Luino (VA) Italy in one single day.

Participants were asked to walk barefoot as normally as possible. Markers (2 cm × 2 cm) were placed at the greater trochanter, head of the fibula and the lateral malleolus in both lower limbs.

Before recordings, subjects walked on the platform until they become familiar with the equipment. Videos were considered for subsequent analysis if at least three steps for each right and left foot were correctly positioned on the WBB while walking.

Finally, the body weight was recorded by the WBB.

Video recordings were divided into segments comprising the left and right stance phases, starting from heel strike until toe-off, as judged by eye and agreed upon by three different viewers. Trajectories over time of the three reference points of each leg were extracted from video recordings using *Tracker Video Analysis and Modeling Tool* (v4.11.0, Open Source Physics project, https://physlets.org/tracker/).

Data points from WBB were acquired with *a custom software*. Due to the WBB design, only vertical GRFs were measured and expressed as Kg_f_. Data processing was performed with *Smalltalk VisualWorks*^*®*^ *v7.10.1* (http://www.cincomsmalltalk.com/main/products/visualworks/) through an ad hoc algorithm for the calculation of the length of the femur, the knee and hip flexion angles and symmetry coefficients.

WBB data and marker points coordinates derived from video frames were both interpolated to 100 points, so to subdivide the whole stance phase in percentiles. A lower GRF threshold of 5 Kg_f_ was arbitrarily set as the stance starting point to exclude possible fluctuations in GRF due to cells not properly loaded.

**Fig. 1:**
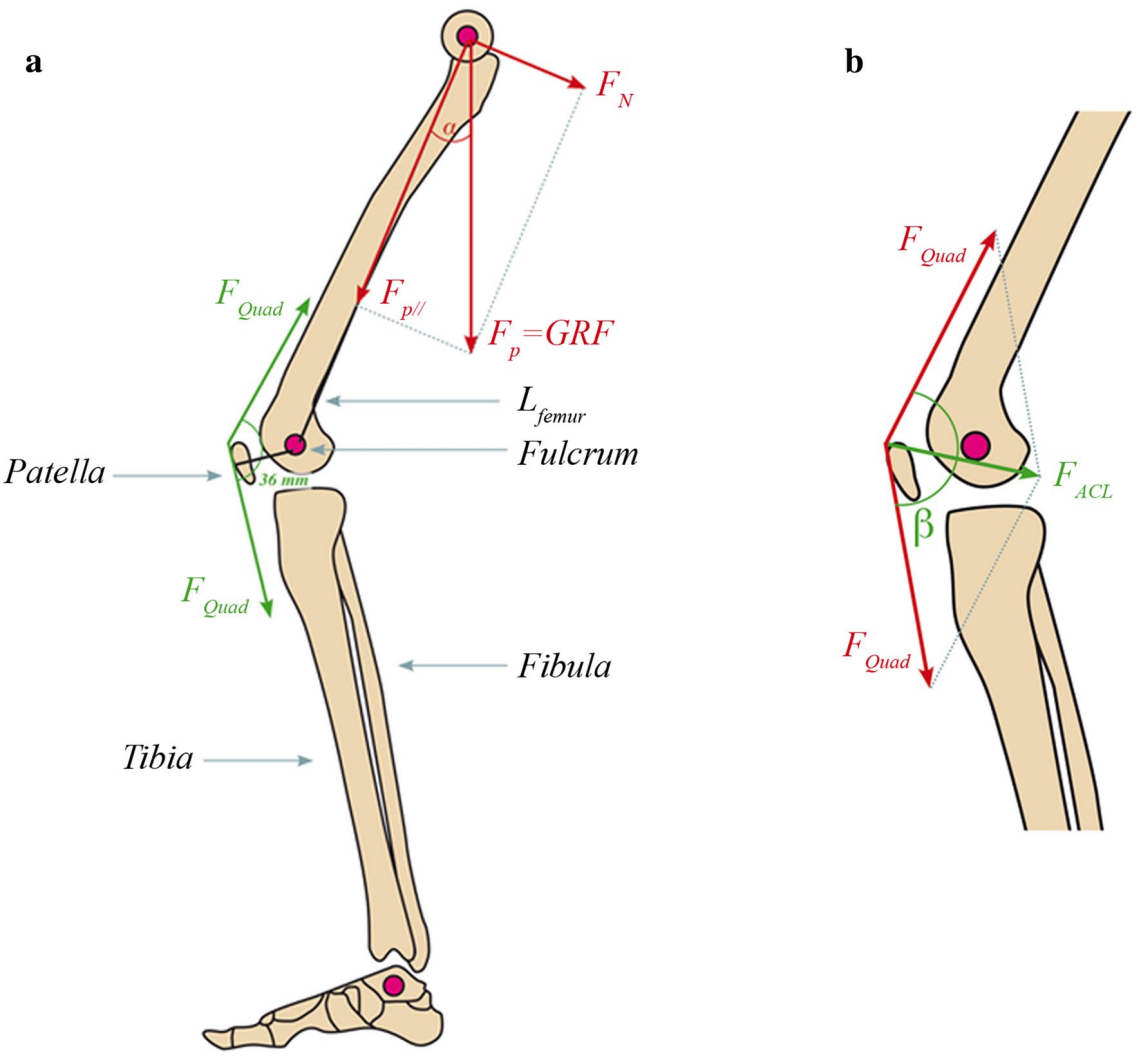
Simplified mechanical models used for computations in the sagittal plane. **a** Decomposition of GRF vector in its longitudinal (F_p//_) and orthogonal (F_N_) components acting at the head of the femur, and the angle that the femur forms with the vertical direction (α) used for the determination of F_quad_. **b** Close-up of the knee joint where, assuming that the patella acts like a pulley, F_quad_ equals the force exerted by the patellar tendon and thus the force acting on the ACL can be computed from the sum of F_quad_ vectors as illustrated, after the measurement of the knee flexion angle (β). Drawing of leg and knee provided by Roberta Frigeri

For each participant, at least three videos and WBB tracks were averaged for each leg. To compute the force exerted by the quadriceps ($F_{Quad}$) at the knee joint the following lever equilibrium condition was considered (Fig. 1a):

$$F_{N} \cdot L_{femur} = F_{Quad} \cdot 0.036$$

where F_N_ is the GRF component normal to the direction of the femur, $L_{femur}$ is the femur length (assuming, in the sagittal plane, that the point of application of $F_{N}$ is at the level of the hip joint) and 0.036 m is a good median estimation of the distance between the tendon, in its frontal passage above the patella, and the center of rotation of the femoral head for shallow knee flexion angles [3]. Given that $\alpha$ is the femur angle with respect to the vertical axis, $F_{N} \, = \,GRF \cdot \sin \alpha$; and resolving for $F_{Quad}$ gives the sought force expressed in Kg_f_.

Using a simplified model [4] of the forces acting at the knee joint (Fig. 1b) the force acting on the anterior cruciate ligament (F_ACL_) was computed as:

$$F_{ACL} = 2 \cdot F_{Quad} \cdot \sin \left( {\beta /2} \right)$$

where $\beta$ is the knee flexion angle.

To quantify the eventual left–right asymmetry of GRF, $F_{Quad}$ and $F_{ACL}$ during the stance phase, symmetry coefficients were computed from GRF, $F_{Quad}$ and $F_{ACL}$ traces as follows [5]:

from the definition of *Overlapping Area* (OA) as

$$OA = \int {\hbox{min} \left( {trace^{r} , trace^{l} } \right)dt}$$

and the *non*-*Overlapping Area* $(OA^{c} )$ as

$$OA^{c} = \int {\hbox{max} \left( {trace^{r} ,\,trace^{l} } \right)dt - OA}$$

it had been possible to distinguish which side gives higher values than the other by defining:

$$OA_{r}^{C} = \int {trace^{r} dt - OA; \quad OA_{l}^{C} = \int {trace^{l} dt - OA} }$$

Then, *Overlapping Coefficient* (OC) was computed as

$$OC = \frac{OA}{{OA + OA_{r}^{C} + OA_{l}^{C} }}$$

giving the percentage of overlap between the two curves. The highest possible OC is 1, indicating perfect symmetry; the calculated lowest OC for F_quad_ is 0.56, and 0.46 for F_ACL_. Each individual will have their OC for GRF, F_quad_ and F_ACL_ assigned to tertiles group (tertiles determined by lowest OC to theoretical highest OC of 1, pooling together all the OC for the three parameters). Individuals in tertile 3 have higher symmetry than those in tertile 1 (i.e. OC closer to 1). Also note that OC for GRF, F_quad_ and F_ACL_ for each individual may be classified into different tertile. Albeit not strictly correct, we intentionally used the highest theoretical value of 1 to implicitly mean that any OC belonging to tertile 3 would have been seen as the closest to perfect symmetry.

To investigate a possible lateral imbalance, we next defined a *Right Symmetry Coefficient* ($SC^{r}$) and a *Left Symmetry Coefficient Left* ($SC^{l}$) as

$$SC^{r} = \frac{{OA_{r}^{C} }}{{OA^{c} }}; \quad SC^{l} = \frac{{OA_{l}^{C} }}{{OA^{c} }}$$

From here, for each subject a coefficient of lateral imbalance was calculated as the absolute difference between $SC^{r}$ and $SC^{l}$ for both F_quad_ and ACL as:

$$\Delta_{Quad} = \left| {SC_{Quad}^{r} - SC_{Quad}^{l} } \right|, \quad \Delta_{ACL} = \left| {SC_{ACL}^{r} - SC_{ACL}^{l} } \right|$$

where a value of zero means perfect lateral symmetry, and 1 complete lateral asymmetry.

Data are expressed as mean ± SEM. Statistics were performed with GraphPad Prism^®^ by the One way ANOVA plus Bonferroni’s multiple comparison test, unless otherwise specified, and statistical significance was set at p < 0.05.

### Results

**Fig. 2:**
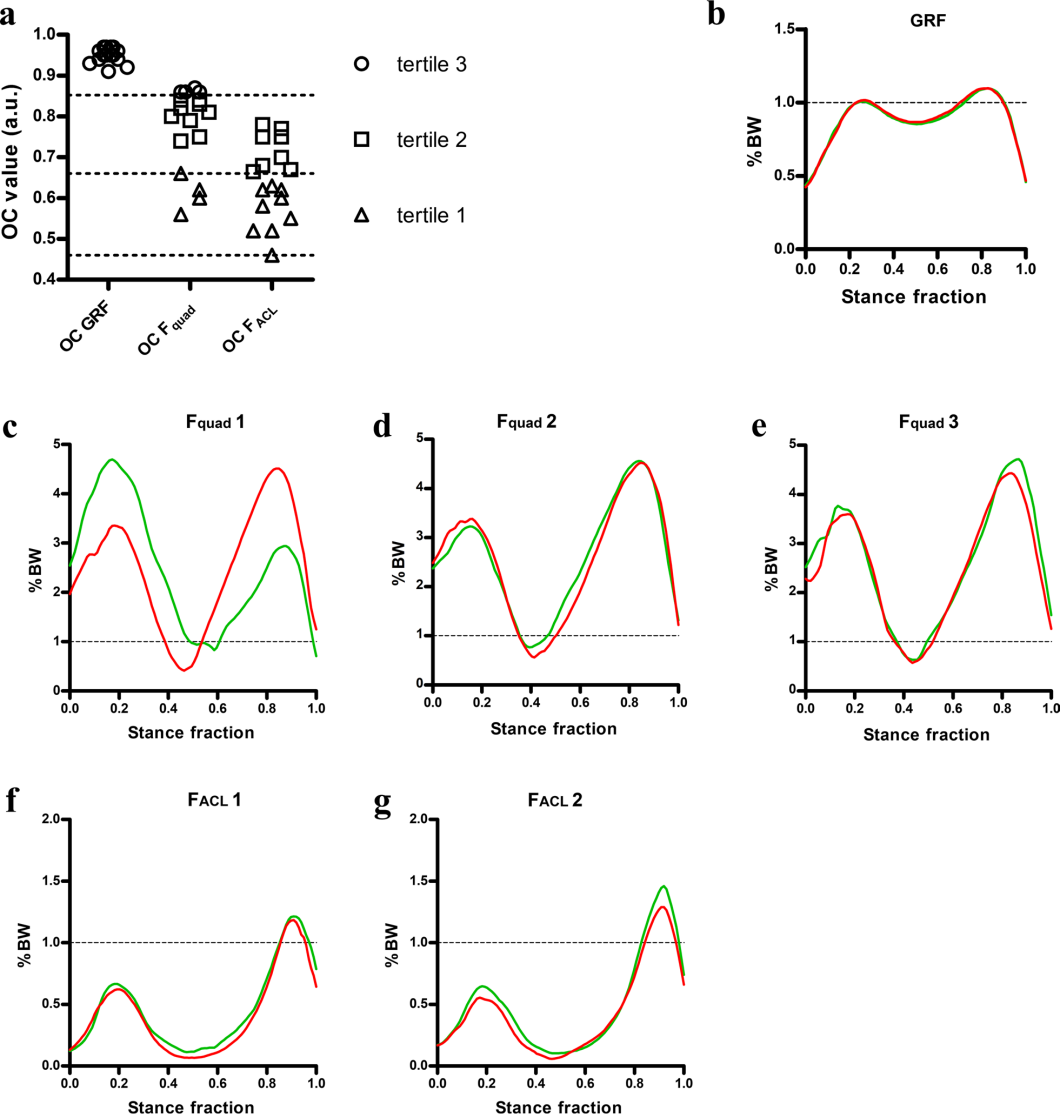
Symmetry coefficient groups and averaged traces. **a** Distribution of GRF, F_quad_ and F_ACL_ OC among groups. Dashed lines indicate boundaries between tertiles.. n = 17 subjects for GRF, 4 for F_quad_ 1, 9 for F_quad_ 2 and 4 for F_quad_ 3; 9 for ACL 1 and 8 for ACL 2. Mean traces of right (green) and left (red) lower limb stance phases for GRF (**b**), F_quad_ (**c**–**e**) and ACL (**f**, **g**). Traces represent the average of the right and left stance phases recorded from the subjects belonging to each tertile. For tertile 1 (lowest OC, worst case, **c**, **f**) it is relevant to note how, notwithstanding a symmetrical GRF (**b**), there is a asymmetry in the load acceptance period (within 0.2–0.3 of the stance phase) and in the subsequent pre-swing period (0.6–0.7 onwards, more evident in **c**). Force (Kg_f_) in **b**–**g** has been normalized to body weight (BW) i.e. a value of 1 means 100% BW. The stance phase (X axis) has been divided into percentiles from 0 (hill strike) to 1 (toe off). Mean traces shown in panels **b**–**g** for left and right legs were obtained by averaging the individual traces of the subjects whose OCs belonged to that tertile. In this respect, the difference between left and right legs might not be obvious by simply looking at the traces (i.e. as in **f**). Numerical indexes of Fquad (1, 2 and 3) and of F_ACL_ (1 and 2) refer to the OC tertile the subjects whose mean traces are plotted belong to. (i.e. **c** shows the mean traces of the subjects whose Fquad OC is in tertile 1)

**Fig. 3:**
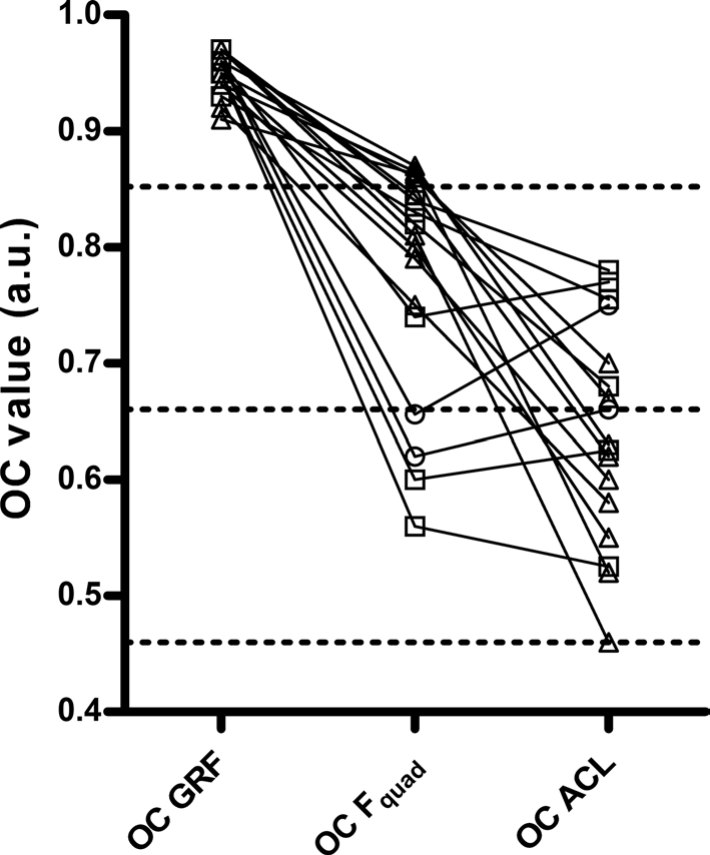
Plots of GRF, F_quad_ and F_ACL_ OC are presented for each subject. A line links the individual OC belonging to the same subject while the symbol shape indicates a shift of the OC from F_quad_ to F_ACL_ towards a lower tertile (up-triangle, n subjects = 9), an upper tertile (circle, n = 2) or the same tertile (square, n = 6)

OCs of GRF all belonged to tertile 3, they were very homogeneous (mean OC 0.95 ± 0.01, n = 17) and close (but significantly different, p < 0.01 n = 17, *One tail Student’s t*-*test*) to unity (Fig. 2a). However, OCs of $F_{Quad}$ calculated from plots of left and right legs forces (Fig. 2c–e) were distributed in all three tertiles (Fig. 2a). while ACL OCs calculated from plots of left and right legs forces (Fig. 2f, g) were only present in tertiles 1 and 2 (Fig. 2a). From the data reported in Fig. 3, among all participants, 53% of them (9/17) lowered the symmetry level from $F_{Quad}$ to ACL (up-triangles), 12% of them (2/17) increased their symmetry level from $F_{Quad}$ to ACL (circle), and the remaining 35% of them (6/17) did not change the level of symmetry when comparing $F_{Quad}$ to ACL (squares).

$SC_{Quad}^{r}$ and $SC_{Quad}^{l}$, of the subjects whose F_quad_ OCs were comprised in tertiles 2 and 3 were even, while subjects whose F_quad_ OCs were in tertile 1 showed an imbalance towards the right leg.

The two ACL groups had $SC_{ACL}^{r}$ and $SC_{ACL}^{l}$ coefficients of about 0.60 and 0.40, respectively, without significant differences among the groups.

However, averaged $\Delta_{Quad}$ (0.27 ± 0.06) and $\Delta_{ACL}$ (0.62 ± 0.08) on the whole population showed a statistically significant (p < 0.01, n = 17, unpaired *Student’s t*-*test*) decreased symmetry of the latter.

### Discussion

Present data were collected from “healthy” young subjects practicing soccer in an agonistic environment. Despite no one reported pain during walking, a clear asymmetry between left and right stance phases emerged with respect to the force of the quadriceps $F_{Quad}$ and the load acting on the ACL (Fig. 2c–g) This phenomenon, which can be ascribed to the asymmetry in hip flexion angle and the added knee flexion angle asymmetry between left and right legs, becomes more evident from $F_{Quad}$ to ACL (see above $\Delta_{Quad}$ and $\Delta_{ACL}$ comparison for the whole population and plot of Fig. 3) and could be regarded as a valuable tool for the discovery of subtle signs of poor gait attitude to be further considered, especially in young athletes.

Literature reports several investigations pointing to the asymmetry of normal gait [6, 7] and fatigue or asymmetrical muscle strength might exacerbate this condition [8]. Moreover, anatomical variability might also be responsible for asymmetrical knee joint kinematics. Nevertheless, this condition might negatively affect the performance in running [9] or even result in injuries in athletes [10]. However, these data have been recorded with professional instruments which are not readily available for routine screening.

Indeed, our work shows that a low-cost assessment of legs kinematic and dynamic asymmetries can be performed even on-field, expanding the possibility to perform a more informative gait analysis to a larger population of normal people and nonprofessional athletes, which could benefit from this.

To this extent, the presently measured difference in peak ACL force (about 25% BW in the worst case belonging to tertile 1) between left and right legs in F_ACL1_ group at heel strike, imposed on the right leg at each step, might result in a larger wear to the right ACL over time.

Moreover, data point to the fact that asymmetries in $F_{Quad}$ and ACL forces might be the result of a complex compensatory mechanism so that the overall, “propriocepted” GRF stays symmetrical during normal gait, as recorded traces (Fig. 2b) and OCs referred to GRF testimony (Fig. 2a). Thus, an almost symmetrical GRF might not always imply a similar symmetry of the underlying forces acting on the lower limbs during normal gait, and if this type of information is needed, a more in deep evaluation should be performed.

Our data show that, by following the recording and analysis procedure outlined in the present work, this deeper evaluation of gait can be achieved at a very low cost and even on-field.

## Limitations

- Limited number of observations.
- Possible artifacts due to the walk of the subjects on a narrow platform.
- Lesser precision than a full-fledged gait analysis system.
- Presently, only sagittal plane investigation.

## Data Availability

The datasets used and/or analyzed during the current study are available from the corresponding author on reasonable request

## Abbreviations

GRF: ground reaction force
ACL: anterior cruciate ligament
WBB: Wii Balance Board
